# Identifying the essential genes of *Mycobacterium avium* subsp. *hominissuis* with Tn-Seq using a rank-based filter procedure

**DOI:** 10.1038/s41598-020-57845-7

**Published:** 2020-01-23

**Authors:** William M. Matern, Robert L. Jenquin, Joel S. Bader, Petros C. Karakousis

**Affiliations:** 1 Department of Biomedical Engineering, Johns Hopkins School of Medicine, Baltimore, MD USA; 2 High-Throughput Biology Center, Johns Hopkins School of Medicine, Baltimore, MD USA; 3 Center for Tuberculosis Research, Division of Infectious Diseases, Johns Hopkins School of Medicine, Baltimore, MD USA; 4 Department of International Health, Johns Hopkins Bloomberg School of Public Health, Baltimore, MD USA

**Keywords:** Bacterial infection, Bacteriology

## Abstract

*Mycobacterium avium* subsp. *hominissuis* (MAH) is increasingly recognized as a significant cause of morbidity, particularly in elderly patients or those with immune deficiency or underlying lung impairment. Disease due to MAH is particularly difficult to treat, often requiring years of antibiotic therapy. Identification of genes essential for MAH growth may lead to novel strategies for improving curative therapy. Here we have generated saturating genome-wide transposon mutant pools in a strain of MAH (MAC109) and developed a novel computational technique for classifying annotated genomic features based on the *in vitro* effect of transposon mutagenesis. Our findings may help guide future genetic and biochemical studies of MAH pathogenesis and aid in the identification of new drugs to improve the treatment of these serious infections.

## Introduction

The genus Mycobacterium contains a variety of difficult-to-treat pathogens frequently associated with pulmonary disease. One of these pathogens, *Mycobacterium avium* subsp. *hominissuis* (MAH), is an opportunistic pathogen associated with significant morbidity in the elderly and in patients with underlying lung disease^1,2^, as well as increased mortality in patients with AIDS^3^. Similar to other mycobacteria, MAH is often difficult to treat effectively with existing antibiotic combinations. Current antibiotic regimens require a median of 5 months to convert the sputum to a culture-negative state^4^, with current guidelines recommending treatment for at least 1 year after sputum culture conversion^5^. Furthermore, a large fraction of patients fail to convert after 1 year of therapy^4^. Patients could greatly benefit from more potent and abbreviated therapies.

Transposon sequencing (e.g., TraDIS^6^, Tn-Seq.^7^, INseq.^8^) has been used extensively to profile haploid genomes and identify gene disruptions that affect bacterial growth under various conditions. Of potential interest in drug development are those drug targets which profoundly disrupt growth on nutrient-rich media (i.e., “essential” genes). In the current study, we have successfully generated genome-wide transposon mutant pools in MAH strain 109 (MAC109). This strain, which was originally isolated from the blood of an AIDS patient, has been characterized extensively in previous studies^9–13^ and is known to infect mice and macrophages^11^. We have utilized the transposon mutant pools we generated to identify genes critical for MAH growth *in vitro* with the goal of informing future research in MAH pathogenesis and drug development. In order to make gene essentiality predictions, we developed a new statistical approach for calling genes based on ranking the read counts from each mutant and applied this to new Tn-Seq data. We report our predictions of the essential genes of MAH and compare these with the predicted set of essential genes in the closely related human pathogen, *Mycobacterium tuberculosis* (Mtb).

## Results

### Constructing genome-wide transposon mutant pools in *Mycobacterium avium* subsp. *hominissuis*

To identify a suitable strain of MAH for genome-wide mutagenesis, we evaluated the ability of the Himar1 transposon (delivered via ΦmycomarT7^14^), which inserts randomly into thymine-adenine dinucleotide (TA sites), to transform common laboratory strains. Transformation efficiency and spontaneous resistance rate (background) were estimated via CFU counts and are provided in Supplementary Table S1. Of the 5 strains tested, MAC109 was observed to have the highest transformation efficiency with only ~1% of untransformed cells being resistant to kanamycin (i.e., ~1% background resistance). Therefore, we decided to proceed with transposon mutagenesis in this strain. Upon transformation, we estimated each of our five independent MAC109 transposon mutant libraries contained between 2.2–4.4 × 10^5^ unique insertion events, for a combined total of 1.2 × 10^6^ unique events with ~2% background resistance. To assist with analysis, we recently provided the genome of this strain, which was found to contain a 5,188,883 bp chromosome and two multi-copy plasmids (pMAC109a and pMAC109b) of lengths 147,100 bp and 16,516 bp, respectively^15^.

### Confirmation of site bias

**Figure 1:**
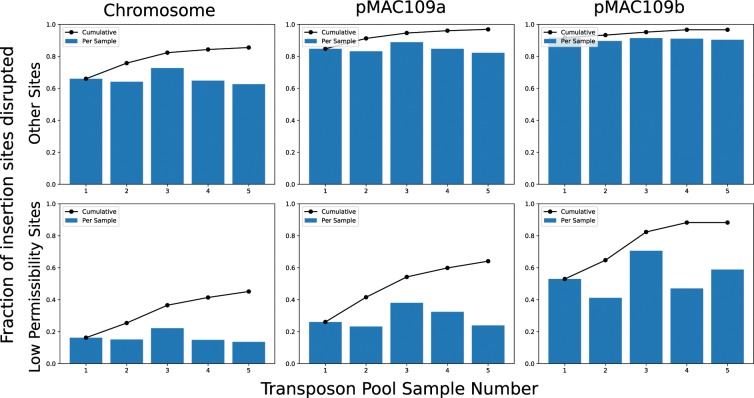
Each barplot shows the fraction of potential Himar1 insertion sites (TA dinucleotide) observed to have sustained at least one insertion in each independent pool of mutants for each replicon of the MAC109 genome. The line plots indicate the cumulative fraction of occupied insertion sites. Notably, the fraction of unique sites occupied saturates for sites not matching the previously defined sequence motif for low permissibility sites ([CG]GNTANC[CG]). However, sites matching this motif can be seen to be near saturation only in the case of the small plasmid (pMAC109b).

The Himar1 transposon/transposase system is known to have a reduced rate of insertion in sites containing the sequence motif [CG]GNTANC[CG]^7^. Indeed, our results confirm that insertion into these low permissibility sites is much less likely than other sites (Fig. 1). Although our approach was able to disrupt nearly all possible insertion sites in the genome not matching this motif (i.e., achieving saturation), a substantial fraction of the low permissibility sites in the chromosome were unoccupied in all five libraries. This effect was less apparent in the plasmids, likely due to their multiple copy number^15^.

### Annotation of MAC109 genetic features

Across our 5 independent libraries we found that 50,203/60,129 (83.5%) sites in the MAC109 genome (including plasmids) could sustain insertions. Our analysis method classified 270 features as ES (Essential), 489 features as GA (leading to a Growth Advantage), and 1267 features as GD (leading to a Growth Defect) out of 5091 total annotated features. 73 features contained no TA sites and 9 features only contained TA sites shared with another feature. Therefore, these 82 features could not be evaluated with the Himar1 system. Our method classified 259 annotated coding sequences, 8 tRNAs, and 2 rRNAs, as well as the only annotated tmRNA as ES. No annotated pseudogenes were labelled as essential, although a minority of them were found to affect growth (i.e., were GA/GD). A summary of classifications by feature type is provided in Table 1, with classifications for individual features provided in Supplementary Table S2. Supplementary Table S3 provides these classifications merged with the raw read count data. Interestingly, our method identified 3 annotated coding sequences in pMAC109a and 1 coding sequence in pMAC109b as essential.

**Table 1:** Table of features annotated by our analysis method.

	CDS	Pseudogene	tRNA	Riboswitch	rRNA	ncRNA	tmRNA
NE	2850	117	8	5	2	1	0
ES	259	0	8	0	2	0	1
GD	1208	32	26	0	0	1	0
GA	460	29	0	0	0	0	0
N/A	64	13	4	1	0	0	0

NE = No Effect, GD = Growth Defect, ES = Essential, GA = Growth Advantage, N/A = Feature lacks potential insertion sites (TA dinucleotide) for the Himar1 transposon or only contains sites shared with another feature. Abbreviations: CDS – coding sequences, RNA – ribonucleic acid, tRNA – transfer RNA, rRNA – ribosomal RNA, ncRNA = non-conding RNA, tmRNA – transfer messenger RNA.

We also compared our method with the TRANSIT Hidden Markov Model (HMM) method developed by Dejesus and colleagues (version 3.0.1)^16^. Using the same raw data, the HMM predicted 282 essential genes (compared to 270 with our novel method). The site-by-site classification as reported using TRANSIT is reported in the final column of Table S3. A Venn diagram showing the number of genes predicted to be essential by each method is reported in Fig. S2. The large overlap and similar number of essential genes between the two methods suggests that their sensitivity and performance are similar at this sample size.

### Comparison of annotations with previously published transposon-based annotations in Mtb

**Figure 2:**
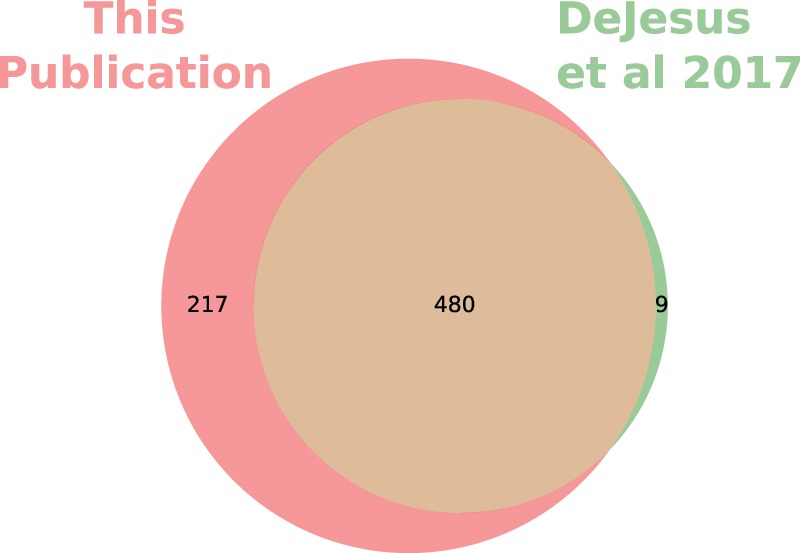
Venn diagram of essential genes predictions for *Mycobacterium tuberculosis* strain H37Rv from our analysis (Supplementary Table S4) compared to the previously published essential gene predictions from DeJesus *et al*.^7^. Notably, the genes labelled essential by the HMM are nearly a subset of the genes labelled as essential by our method. Only protein coding sequences are considered in this diagram.

We applied our analysis method to previously published Tn-Seq data^7^ and compared the results of our novel analysis method (Supplementary Table S4) with the results of a previous analysis method^7^, which utilized a HMM to detect essential genes. All genes labelled as “ESD” (containing an essential domain) by the previous analysis method were considered ES for comparison. Figure 2 shows the overlap in the predicted essential coding sequences (CDS) from each method (RNA and other features excluded). Overall, there was good agreement between each method, although our method appears to be somewhat more sensitive for essential gene detection than the previous method at this sample size. Of note, the essential genes unique to our method contained a significant number of sites with zero or very few insertions, but these sites were interspersed among sites containing larger numbers of reads. This is consistent with expectations that the HMM used previously is sensitive primarily to multiple adjacent sites with low read counts, whereas our method is sensitive to the number of sites per gene, regardless of position.

## Discussion

Using our novel computational method we identified 230 genes as essential in both MAH and Mtb (Supplementary Table S5). These may represent particularly good targets for drug development, as inhibitors of a gene product are likely to be effective against a close ortholog. As expected, a number of well-demonstrated targets are present, including the targets of the mycobacterial drugs cycloserine (alanine racemase, D-alanine – D-alanine ligase), rifamycins (RNA polymerase beta subunit), macrolides (50 S ribosome), aminoglycosides (30 S ribosome), fluoroquinolones (type IV topoisomerases and gyrases), bedaquiline (ATP synthase), and ethambutol (arabinosyltransferase). Additional compounds that have been reported to have some activity against mycobacteria include tryptophan synthase inhibitors^17^, ClpP inhibitors^18^, and Rho inhibitors (albeit only shown to be effective through genetic manipulation)^19^. A brief literature search also reveals many compounds that inhibit non-mycobacterial orthologs of these gene products, but data are lacking on their killing activity against mycobacteria, including inhibitors of GroEL^20^, RibBA^21^, SecA^22^, and LigA^23,24^. Thus, many opportunities are available for targeting the products of these overlapping essential mycobacterial genes.

Our analysis classified four protein-coding genes on the two plasmids as essential (3 on pMAC109a and 1 on pMAC109b). In previous work, we showed that strain MAC109 contained plasmids pMAC109a and pMAC109b with copy numbers of 1.76x and 4.92x, respectively^15^. We theorized that a plasmid with a high copy number should be able to sustain transposon insertions in a gene (or any trans-acting element) without impact on plasmid survival due to complementation by other copies of the plasmid. Thus, it is surprising to identify an essential gene in pMAC109b. On the other hand, given the much lower copy number of pMAC109a, genes required for replication of this plasmid might be expected to be annotated as essential.

We used NCBI BLAST to find homologs of the genes identified as essential in the plasmids. DFS55_24645 (on pMAC109a) and DFS55_25425 (on pMAC109b) are homologous to Rep, a protein critical for the replications of plasmids. Thus, one possible explanation for the essentiality of these Rep homologs is that plasmid copy number will decrease in daughter cells inheriting the plasmid (with no plasmid replication possible in a cell with all copies containing disrupted Rep). This is a strong selective pressure against the mutant plasmid. DFS55_14680 (on pMAC109a) is a ParA homolog. ParA controls the distribution of plasmids to daughter cells such that cells inherit the plasmid more equally. It is not immediately apparent how a more random distribution of the plasmids due to disruption of ParA would lead to a growth defect. Lastly, DFS55_24600 (on pMAC109a) is a hypothetical protein also classified as essential. It lacks a paralog in the MAC109 chromosome and an ortholog in Mycobacterium avium strain 104 (which does not contain plasmids). Thus, it appears to be non-essential for the MAH pangenome. DFS55_24600 is homologous to Rv3081 from H37Rv and our analysis identified Rv3081 as “GD” (approximately 0.25 relative fitness). It is also apparent from examining the raw Tn-seq read counts (Supplementary Table S3) that transposon insertion in the beginning of this gene does not have a profound effect on growth rate in MAC109 (this trend is less clear in H37Rv). Future work could clone DFS55_24600 into an episomal (non-integrating) mycobacterial shuttle vector (such as pPB10) and examine the retention of the episome with and without this gene in the absence of antibiotic selection. Additionally, an attempt could be made to isolate a MAC109 mutant cured of pMAC109a.

During the review of this manuscript, a study by Dragset *et al*.^25^ was published describing, in part, the characterization of essential genes of a different strain of MAH using transposon mutagenesis. Interestingly, they observed transposon insertions in 36813 of 55516 TA sites on the chromosome (66.3%) whereas we observed insertions in 47790 of 57588 TA sites (83%). This difference in the number of TA sites occupied is reflected in the number of essential genes identified in each of these two studies. Dragset *et al*. identified 362 out of 4653 genes (7.8%) as essential for *in vitro* growth. On the other hand, when applying the same TRANSIT HMM algorithm, we identified 282 out of 5091 (5.5%) genomic features as essential. This difference suggests that some of the genes previously labeled as essential may not be broadly essential for *in vitro* growth. The discrepancy may reflect methodological differences. Dragset *et al*. generated approximately 170,000 independent transformants across two independent transformations, whereas we generated ~7-fold more (~1,200,000) transformants across 5 independent transformations, greatly improving the probability of obtaining transposon insertions in nonessential genes. The higher fraction of occupied TA sites in our study also may reflect our use of a more permissive growth medium (7H11 vs 7H10 agar). Finally, the two studies also utilized different strains of MAH (MAC109 vs MAH 11), which we expect to be a minor factor given their close genetic similarity^25^.

Our analysis method has several advantages over other methods, including its anticipated robustness based on use of the zero-inflated negative-binomial to model read counts, which can more accurately account for non-saturating libraries, as these have a high probability of containing sites without observed insertions. This may be especially important for transposons which cannot easily achieve saturation without very large numbers of transformants (due to lack of the strict TA site bias of Himar1), such as the Tn5 system^26^. Also, we have fully exploited the statistical independence of samples, which increases our statistical power. Other models, such as hidden Markov models, generally pool samples, limiting the usefulness of having biological replicates.

However, our method also has limitations. Using our collected data, we detected a somewhat low number of essential features after applying our method to data from MAC109 relative to H37Rv (270 and 738 ES by applying our method to both datasets, respectively) despite evidence that the genome was saturated with insertions (Fig. 1). Most likely, this is due to our somewhat low sample size (5 independent libraries) in MAC109 versus H37Rv (14 independent libraries). Therefore, we believe that sequencing additional independent transposon mutant libraries could significantly increase the statistical power to detect essential genes in MAC109, particularly for features with fewer insertion sites. Additionally, while our method can correctly handle known sites with low rates of insertion (e.g., [CG]GNTANC[CG]), it is possible that additional such sites exist that have not yet been defined. Identifying sites with low rates of insertion is especially important to avoid false positives (features falsely classified as essential).

In conclusion, we have generated genome-wide transposon mutant pools in MAH strain MAC109, collected sequencing data, and used a novel approach for annotating genes based on these data. We find that these pools are nearly saturated with transposon insertions except at low permissibility sites previously shown to have a reduced insertion rate. Our analysis identified the essential genes of MAC109 and we suggest explanations for the apparent detection of essential genes in the plasmids. Further characterization of independent MAC109 transposon mutant libraries could increase the sensitivity of detecting essential genes. Future work could validate our high-throughput predictions by adapting the existing mycobacterial dCas9 knockdown system^27^ to MAH and measuring the impact of individual gene knockdown on bacterial growth rate.

## Materials and Methods

### Strains

MAH strains MAC109, MAC104, and OSU3388 were a gift from Dr. Luiz Bermudez (Oregon State University). MAH strain MAC101 (Chester, ATCC 700898) was a gift from Dr. Eric Nuermberger (Johns Hopkins School of Medicine). Individual colonies of each strain were isolated and regrown to make stocks used in the described experiments. MAC101 was seen to form both translucent and opaque colonies. Both an opaque (MAC101o) and a translucent (MAC101t) colony were isolated and used for stocks.

ΦmycomarT7 was propagated and titered as previously described^28^. Final titers used for transformations exceeded 10^11^ PFUs/mL.

### Media and buffers

To make 7H11 agar 10.25 grams of 7H11 w/o Malachite Green powder (HiMedia Cat No. 511 A) was added to 450 mL deionized water. 5 mL 50% glycerol was then added before autoclaving. Hot agar was cooled to 55 °C before addition of 50 mL OADC enrichment and 1.25 mL 20% Tween-80.

To make 7H9/10% OADC: 2.35 g 7H9 powder was added to 450 mL deionized water. After sterilization (via autoclaving at 121 °C or by passing through a 0.22 um filter) 50 mL of OADC enrichment (Becton-Dickinson) was added. Unless otherwise specified, no Tween-80 or glycerol was included.

To make 7H9/50% OADC, the protocol was identical to that for 7H9/10% OADC, except 250 mL water was added to 250 mL OADC. To make PBS-Tw, 1.25 mL filter-sterilized 20% Tween-80 was added to 500 mL sterile PBS. The MP Buffer consisted of 50 mM Tris-HCl (pH 7.5), 150 mM NaCl, 10 mM MgSO_4_, and 2 mM CaCl_2_. In each case, individual components were autoclaved prior to mixing in solution.

### Testing of transformation efficiency of MAH strains

Five strains of MAH (MAC109, MAC104, OSU3388, MAC101o, MAC101t) were tested for transformation by ΦmycomarT7. For transformation, strains were grown in 150 mL of 7H9/10% OADC. After OD of each strain reached 0.32–0.89, 100 mL of cultures were equally split into two 50 mL conical tubes. Bacteria were pelleted via centrifugation and resuspended in 10 mL MP buffer. Bacteria were pelleted again via centrifugation and resuspended in 4.5 mL MP Buffer. 0.5 mL of MP Buffer (negative control) or ΦmycomarT7 stock (approximately 10:1, phage:bacteria) was added to each tube. Tubes were incubated for two days shaking at 37 °C. Bacteria were then pelleted via centrifugation and resuspended in PBS-Tw (phosphate-buffered saline containing 0.05% Tween-80). Bacteria were then pelleted again and resuspended in 1 mL of PBS-Tw. Transformed bacteria and negative control for each strain were then diluted in PBS-Tw and plated on 7H11 with and without 50ug/mL kanamycin for titration. As this assay was done to quickly identify a transformable strain, only a single replicate was performed for each strain. Therefore, no standard deviation or mean is reported in Table S1.

### Generation of transposon mutant libraries in MAC109

In preliminary experiments, we found that MAC109 growth increased at higher concentrations of OADC. We suspect the oleic acid in OADC is the key to achieving this, based on previous reports^29^. Five independent transposon mutant pools were generated. MAC109 was grown in 700 mL 7H9/50%OADC to OD 2.1 in two 1.5 L roller bottles shaking at 37 °C. Based on previous results (data not shown) we estimated the initial bacterial density based on optical density to be 4 × 10^8^ CFUs/mL for calculation of volume of phage stocks. Bacteria were aliquoted to 12–50 mL conical tubes and centrifuged (2000g for 5 minutes) and supernatant removed. 5 mL MP Buffer was added to each tube and bacterial pellet was resuspended. Pairs of tubes were pooled yielding 6–10 mL aliquots. Samples were then centrifuged (2000g for 5 minutes) and the supernatant was removed. Phage (10:1, phage:bacteria) was then added to all tubes except for the no-vector control. MP Buffer was added to all tubes to a final volume of 5 mL and bacterial pellets were dispersed via pipette. Bacterial/phage mixtures were then placed on a shaker incubator (37 °C) for two days. Tubes were then centrifuged (2000g for 5 minutes) and supernatant was removed. Ten mL PBS-Tw was then added and the bacterial pellet was dispersed via pipette. Tubes were then spun down again (2000g for 5 minutes), supernatant removed, and 1 mL of PBS-Tw was used to resuspend pellets.

Fifty μL of each tube of washed transformants (or no-vector control) were diluted and plated on 7H11 plates, with or without 50ug/mL kanamycin, to determine transformation efficiency and background resistance. The remainder of the cultures were plated on 7H11 containing 50ug/mL kanamycin in Pyrex baking dishes (15″ × 10″, 500 mL agar per dish, 1 tube per dish). After 7–10 days, colonies were scraped from each dish and dispersed in fresh 7H9 broth and frozen in aliquots at −80 °C for later use.

DNA was extracted from one aliquot of each transposon mutant pool using a previously described gDNA extraction protocol for short read sequencing^15^. We adapted a previously published library prep protocol^30^ to prepare libraries for sequencing. Adaptations include the use of magnetic beads for purification and library size selection as well as changes to PCR conditions (for details see Supplementary Text S1). Libraries were sequenced (2 × 75 bp) on an Illumina HiSeq. 2500 by the Johns Hopkins GRCF High Throughput Sequencing Center. 5 independent libraries were sequenced yielding between 2,194,085–4,381,545 reads per library for a total of 18,197,728 paired-end reads.

### Raw data processing

We previously showed that the MAC109 genome contains two plasmids in addition to the bacterial chromosome^15^. We adapted the TRANSIT pre-processor (tpp)^16^ to allow for mapping to multiple contigs. These changes were included in the release of TRANSIT/tpp v2.4.1. We used tpp v2.4.1 to map all reads to the MAC109 genome. The command for processing raw reads was: tpp -himar1 -bwa -bwa-alg aln -ref MAC109.gb -replicon-ids “CP029332,CP029333,CP029334” -reads1 TnPool_1.fastq -reads2 TnPool_2.fastq -window-size 6 -primer AACCTGTTA -mismatches 2. CP029332, CP029333, and CP029334 are the Genbank identifiers for the chromosome and two plasmids, respectively, as required by tpp. After PCR duplicate removal, a total of 10,597,261 unique reads mapped to the genome and were used for analysis.

### Statistical analysis

We use a previously suggested labelling scheme^31^ to annotate each gene of MAC109. A gene is labelled NE (No Effect) if a transposon insertion in any of its potential insertion sites causes no effect on growth. A gene is labelled GD (Growth Defect) if it contains at least one insertion site such that upon transposon insertion it results in a decrease in bacterial growth. A gene is labelled GA (Growth Advantage) if it contains at least one insertion site such that upon transposon insertion it results in an increase in bacterial growth. A gene is labelled ES (essential) if it contains at least one insertion site such that upon transposon insertion it results in a large loss in viability.

To annotate the MAC109 genome, we have designed a robust procedure. Some additional details of this method are provided in the supplement (Supplementary Text S2). At a conceptual level, our analysis pipeline proceeds in two steps. First, insertion sites without a growth defect are approximately identified with a rank-based filter procedure. Second, the counts at the insertion sites identified by the filter are assumed to approximate the null distribution and used for statistical hypothesis testing. For identification of ES genes, the approximate null distribution is fit to a zero-inflated negative binomial distribution (using maximum likelihood estimation) which is then scaled and used for hypothesis testing. For identifying the GD and GA sites, the empirical cumulative distribution function is used for hypothesis testing. Stouffer’s method is used to combine p-values from multiple replicates and multiple sites. Lastly, multiple hypothesis correction is performed (Benjamini-Hochberg for ES, Bonferroni for GD/GA testing). The less conservative, FDR-style Benjamini-Hochberg procedure was paired with the ES test due to its substantially lower statistical power compared to the GD/GA test.

#### Relative fitness

The fitness, relative to wildtype, resulting from disruption of a particular gene is approximated as follows. First, the mean of the read counts at each insertion site is calculated across samples. The site fitness is calculated as the mean read count of each site divided by the median across all sites (i.e., samples are normalized to the median). Finally, each gene is assigned a Relative Fitness equal to the median of the site fitness for all sites contained in the gene.

#### Rank-based filter procedure

We assumed that all mutants with a transposon insertion at the same site will have identical growth rates (i.e., the growth rate is entirely defined by the insertion site). We also assumed that not more than 40% of insertion mutants would have a growth defect and not more than 15% of mutants would have a growth advantage (and therefore at least 45% of mutants would have a growth rate that is identical to wildtype). We selected these thresholds based on previous predictions in *Mycobacterium tuberculosis*^7^ suggesting that 15% of insertion sites cause a growth defect and 8% cause a growth advantage. We have added a large margin of error to ensure conservatism.

Note that if some of the identities of insertions mutants with growth rates identical to wildtype were known ahead of time we could simply use the distribution of the reads at these sites to train a null model to test the other sites. This is the intuition behind our rank-based filter procedure. However, as the identities of the insertion sites with no effect on growth rate are unknown we use an approximation. For each of *J* transposon pools (replicates) we compute the rank of the read count at each site (averaging identical ranks) in the other *J*-1 samples. For each site, we then take the average of these *J*-1 ranks across samples. Lastly, we order the average rank from least to greatest and remove the smallest 40% and greatest 15% (removing additional sites with ties at the threshold), leaving only ~45% of the original insertion sites. The read counts from these remaining ~45% of sites will be distributed approximately the same as an insertion site with no effect on growth. Additionally, previous literature suggests that the Himar1 transposon is biased against insertion sites with the motif (GC)GNTANC(GC)^7^. Therefore, we separately apply the above rank-based filter to the read count data collected from these sites.

To demonstrate the correctness of our rank-based filter procedure we utilized simulated data. Briefly, read counts from 39,000 insertion mutants without a defect were simulated as a negative binomial distribution with mean 35 and dispersion 3.0. These parameters are roughly those found by fitting real data (fitting procedure described below). Additionally, read counts from 15,000 mutants with a growth defect were simulated with a mean of between 0 and 0.67 times that of a no defect mutant using the negative binomial distribution with an identical dispersion. The mean multiplier was chosen for these mutants by uniform sampling between these bounds. Lastly, read counts from 6,000 mutants with a growth advantage were simulated using 1.5 to 4 times the null mean (uniformly distributed) and identical dispersion. Combining these 3 groups of samples provided a simulated transposon mutant library. 5 and 50 independent simulated transposon mutant libraries were generated. The rank-based filter procedure described above was then applied to the resulting datasets. Q-q plots provided in Supplementary Fig. S1B,D comparing the theoretical distribution to the unfiltered and filtered empirical cdfs show that the filter procedure improves accuracy. Increased sample size also improves accuracy, as expected.

#### Hypothesis Testing for Essentiality (ES)

To classify a gene as ES, we performed statistical hypothesis testing. The read counts from the insertion sites identified by the rank-based filter are used to fit a zero-inflated negative binomial distribution (See Supplementary Text S2 for definition). Fitting is done by maximizing the likelihood with L-BFGS-B as implemented in scipy.optimize (Scipy v1.2.1). Using the fit distribution, we then create a new “borderline ES” distribution by scaling the mean of the negative binomial distribution to 5% of the original, keeping the dispersion and zero inflation component constant. We use this borderline distribution to do statistical hypothesis testing on the read counts from each of the sites using the lower tail probability as the p-value. This means that a gene whose insertion gives 5% of WT growth is unlikely to be called ES. While the particular threshold we have chosen (5% of wildtype growth) is somewhat arbitrary, we feel it is both small enough to ensure mutants labelled ES are highly defective but not so small so as to have no hope of classifying highly defective mutants as ES.

To pool essential p-values across samples, we used the one-tailed Stouffer’s method at each site. To pool p-values across insertion sites within a gene we use the truncated product method^32^ with a truncation threshold of 0.5 (τ < 0.5). TPM provides a principled approach for limiting the effect of sites with no associated growth defect which would otherwise greatly inflate the p-values (such as those sites at the C-terminus of the gene which may not disrupt the function of the protein). We then control the False Discovery Rate (FDR) using the Benjamini-Hochberg procedure (FDR < 0.01).

#### GD/GA hypothesis testing

To classify a gene as GD or GA, we performed statistical hypothesis testing. We utilized the read counts for insertion sites identified by the rank-based filter to form an approximate null distribution and used the empirical cumulative distribution function (ecdf) to compute p-values. We generated a separate ecdf for low permissibility sites. We also generated separate ecdfs for each contig as sequencing depth varied greatly between contigs (due to multiple copy-number plasmids). The exact p-value computation, which ensures p-values are continuously distributed, is described in detail in the supplement. For a particular insertion site, the p-values from each sample were pooled using the one-tailed Stouffer’s method. The resulting pooled p-values from all insertion sites within the same gene were then pooled using the two-tailed Stouffer’s method. For declaring genes as GA or GD we set the p-value threshold to allow only a single (expected) false discovery after 5009 tests, corresponding to a single-test p-value of approximately 0.0002. A gene was declared GD if its Relative Fitness was less than 2/3 and was statistically significant (p < 0.0002). Similarly, a gene was declared GA if its Relative Fitness was greater than 1.5 and was statistically significant at the same threshold. Note that if a gene meets the criteria for both the GD and ES label then it is given the ES label only. If it meets the ES criteria but not the GD label it is given the NE label. This can very occasionally happen as a result of the different statistical distributions used to call GD genes and ES genes (empirical null vs negative binomial distribution, respectively, as well as the different procedures for multiple hypothesis correction).

## Data availability

We have made efforts to enable others to reproduce the major results of this paper from the raw data. Scripts and instructions for use are provided at GitHub (https://github.com/joelbader/essential_genes)^33^. Raw data are provided in NCBI’s SRA under accession number: PRJNA527645.

## Supplementary information

Supplementary Figures and Text.

Table S1.

Table S2.

Table S3.

Table S4.

Table S5.
