# Supplementary material for: Identifying the essential genes of Mycobacterium avium subsp. hominissuis with Tn-Seq using a rank-based filter procedure
Source: Sci Rep. 2020 Jan 23;10:1095. doi: 10.1038/s41598-020-57845-7 (PMC6978383; doi:10.1038/s41598-020-57845-7)
Supplement: Supplementary file 1 — Supplementary Figures and Text. [file 41598_2020_57845_MOESM1_ESM.pdf]

Supplementary Info for:

Identifying the essential genes of *Mycobacterium avium* subsp. *hominissuis* with Tn-Seq using a rank-based filter procedure

Running Title: Essential Genes of *Mycobacterium avium*

Authors: William M. Matern[a,b,c], Robert L. Jenquin[c], Joel S. Bader[a,b,c#], Petros C. Karakousis[c,d#]

Affiliations:

a – Department of Biomedical Engineering, Johns Hopkins School of Medicine, Baltimore, MD

b – High-Throughput Biology Center, Johns Hopkins School of Medicine, Baltimore, MD

c – Center for Tuberculosis Research, Division of Infectious Diseases, Johns Hopkins School of Medicine, Baltimore, MD

d – Department of International Health, Johns Hopkins Bloomberg School of Public Health, Baltimore, MD

# - joel.bader@jhu.edu

# - petros@jhmi.edu

Descriptions of supplementary files:

- Supplementary Figure S1: Simulated data showing correctness of rank-based filter.
- Supplementary Figure S2 - Venn diagram of essential gene predictions for MAC109 by our method vs. TRANSIT.
- Supplementary Text S1: Protocol for preparing sequencing libraries
- Supplementary Text S2: Additional details of analysis method
- Supplementary Table S1: Transformation efficiency of avium strains.
- Supplementary Table S2: Gene prediction in MAC109 (with p-values and LFC)
- Supplementary Table S3: Raw data in MAC109 along with gene predictions
- Supplementary Table S4: Essential genes in H37Rv based on previous data
- Supplementary Table S5: Overlap between MAC109 and Mtb essential genes (both computed with our analysis method)

Figure S1

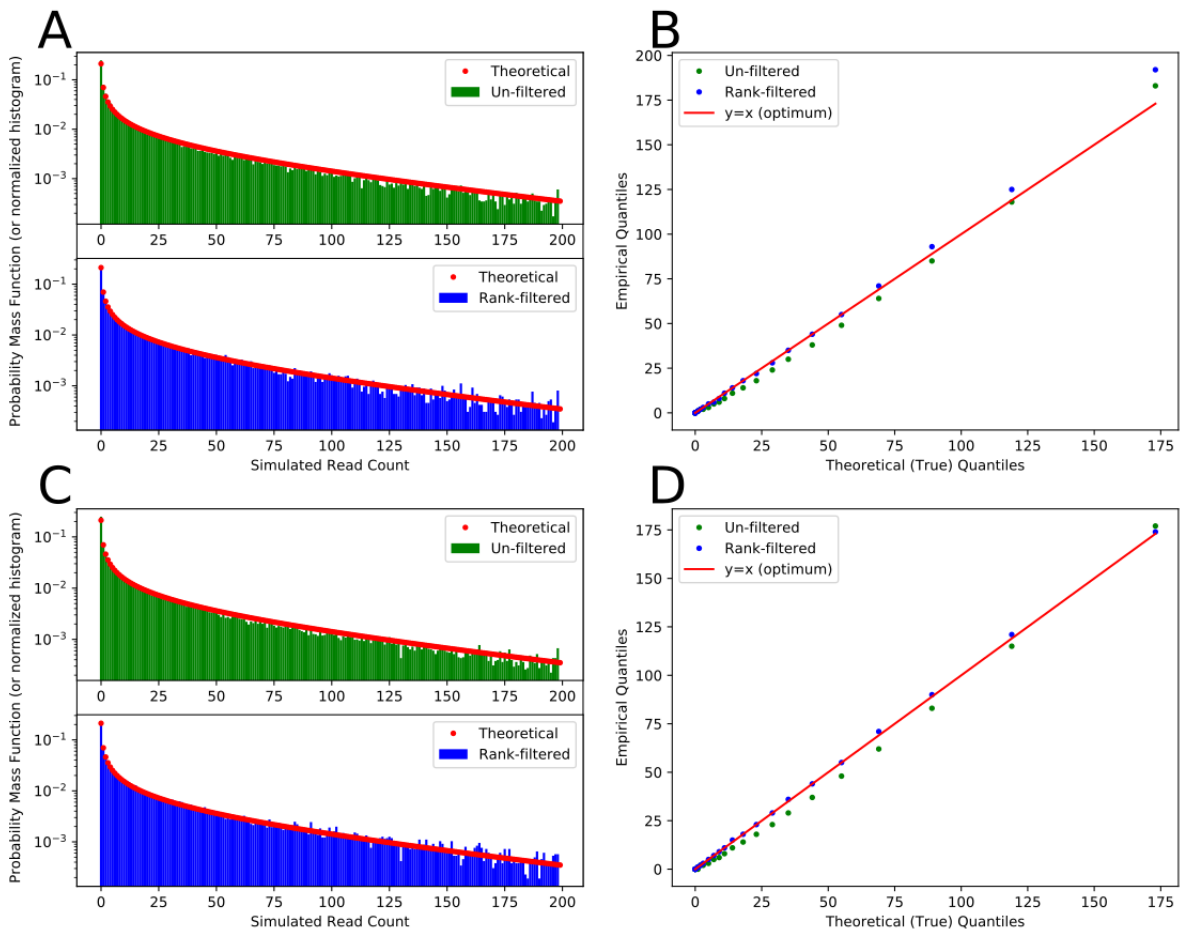

Figure S1: Simulated data showing correctness of rank-based filter. As described, simulated read counts were generated to test our rank-based filter procedure. A simulation of either 5 sequenced samples ((A) and (B)) or 50 samples ((C) and (D)) was generated. (A) and (C) show histograms of the read counts across all sites before applying the filter procedure in green and the read counts after applying the filter procedure in blue. In red we plot the pmf of our sampling distribution for the null distributed sites. Performance was assessed by q-q plots in (B) and (D). In green are the empirical quantiles before applying the rank-based filter procedure and in blue are the quantiles after filtering. The red line represents perfect theoretical performance.

Figure S2

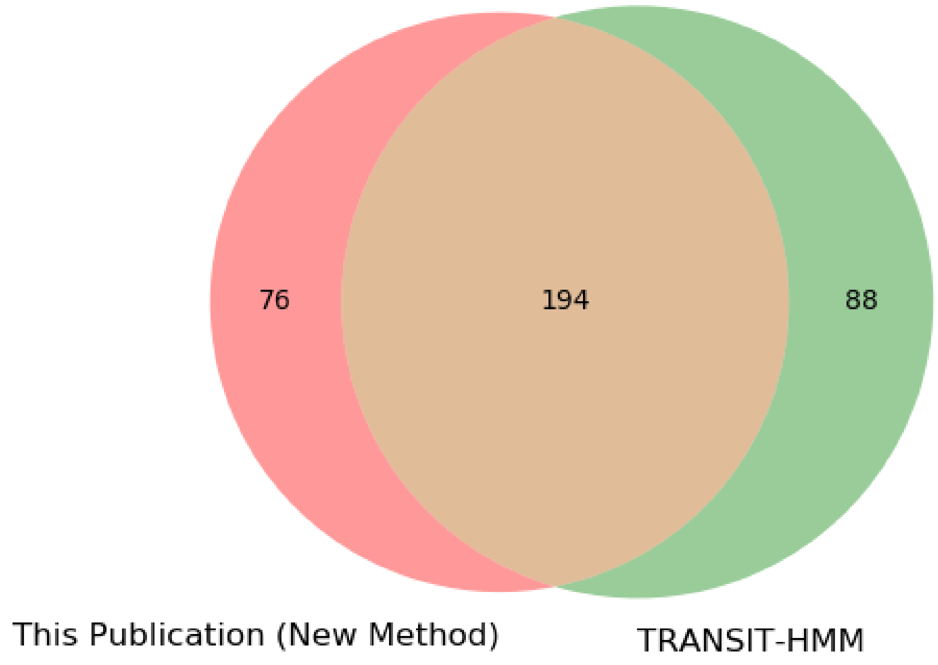

Figure S2 - Venn diagram of essential gene predictions for MAC109 by our method vs. TRANSIT.

Tn-seq Library Preparation-Q5

Version 14

Short Code: tnlibV14

Prepared by: Will Matern

Last updated: 10/01/2019 at 01:57

Equipment and Reagents

- TE (Tris EDTA) buffer
- Covaris MicroTube
- Covaris S220
- Tris-Cl buffer
- Nanodrop
- Thermo-Fisher Fast DNA End Repair Kit (Cat. #K0771)
- AxyPrep beads
- Magnetic plate (for removing beads)
- 70% EtOH (Ethanol)
- ThermoPol buffer
- dNTPs (NEB)
- Taq DNA Polymerase (NEB #M0267)
- 100uM adapter oligo 1 (ATGATGGCCGGTGGATTTGTGNNANNANNNTGGTCGTG-GTAT)
- 100uM adapter oligo 2 (pTACCACGACCA-NH_2, 5 prime phosphorylated with amino modifier at 3 prime)
- 50mM $MgCl_2$
- Thermocycler
- Blunt/TA Ligase Master Mix (NEB #M0367S)
- Q5 Hot Start 2X Master Mix
- 10uM adapter primer (ATGATGGCCGGTGGATTTGTG)
- 10uM transposon primer (TAATACGACTCACTATAGGGTCTAGAG)
- SPRIselect beads
- 85% EtOH
- 10uM sol_adapt [matches adapter, see below for sequence]
- 10uM sol_mar mix [matches transposon, see below for sequence]

Sequencing Oligos

10uM sol_mar mix consists of an equal parts mix of the following oligos diluted to 10uM in Tris-Cl:

- AATGATACGGCGACCACCGAGATCTACACTCTTTCCCTACACGACGCTC TTCCGATCT CGGGGACTTATCAGCCAACC
- AATGATACGGCGACCACCGAGATCTACACTCTTTCCCTACACGACGCTC TTCCGATCT TCGGGGACTTATCAGCCAACC
- AATGATACGGCGACCACCGAGATCTACACTCTTTCCCTACACGACGCTC TTCCGATCT GATACGGGGACTTATCAGCCAACC
- AATGATACGGCGACCACCGAGATCTACACTCTTTCCCTACACGACGCTC TTCCGATCT ATCTACGGGGACTTATCAGCCAACC

10uM sol_adapt consits of *one* of the following oligos diluted to 10uM in Tris-Cl. Note that if multiplexing, a different oligo for each sample on the run must be used. Each oligo is constructed as one of four "backbones" with a different barcode (index) for distinguishing multiplexed samples. A mutliplexed run should consist of an equal molar mix of each backbone (to increase base diversity)

- Backbone 1: CAAGCAGAAGACGGCATACGAGAT XXX XXX XX GTGACTG GAGTTCAGACGTGTGCTCTTCCGATCTGTCAATGATGGCCGGTGGATT TGTG
- Backbone 2: CAAGCAGAAGACGGCATACGAGAT XXX XXX XX GTGACTG GAGTTCAGACGTGTGCTCTTCCGATCTCGTCCATGATGGCCGGTGGAT TTGTG
- Backbone 3: CAAGCAGAAGACGGCATACGAGAT XXX XXX XX GTGACTG GAGTTCAGACGTGTGCTCTTCCGATCTACAGTCCCATGATGGCCGGTG GATTTGTG
- Backbone 4: CAAGCAGAAGACGGCATACGAGAT XXX XXX XX GTGACTG GAGTTCAGACGTGTGCTCTTCCGATCTTAGTGGATGATGGCCGGTGGA TTTGTG

Where "XXX XXX XX" are barcodes used to distinguish mutliplexed samples. Barcodes we've used include the following: "ACA CGA TC", "AGC ATA CA", "TGC TAC GC", "AGT CTA CA", "CTC ATG CA", "AGT TCG GA", "CAT GAT CG", "CGT CAT CA", "CGC GCG GT", "GAC CTG CA", "TGA GAC TT", "AAG TAG AG", "GAG ATC TT", "GCC GAT GT", "TAC GTA CC", "CAG TTC AT", "TCC CTA TA", "GTC CGA TC", "GGT TCA AC", "CAC GTA CT", "TGT CAA GT", "TGT TCC GA", "TTC CGG AG", "CGA TCA AG", "CGA GGA GA", "TGG GGG AC", "TGC CTC GG", "TTA CAA CG", "CGA AAC CC", "ATC ACT CT", "TTC AGC AT", "ACT TGG TG". Note: The Illumina software to demultiplex will organize reads by the reverse complement of the sequence included in the above oligos.

Procedure

Shear DNA

1. Combine 5 ug (or more) of transposon containing genomic DNA with TE buffer to a total volume of 130uL in a Covaris MicroTube.
2. Settings for Covaris S220/E220 in NGSC for shearing to 500bp Note: These differ from settings suggested by Covaris. You should titrate the shear duration the first time and run a gel to confirm proper size. Duty Cycle-5%, Intensity-3, Cycle/Burst-200, Time-50 seconds. Degas water bath for at least 30 minutes. Bath temperature should reach ∼5°C before shearing.
3. Purify DNA with spin column. **Elute into 43.5 uL of Tris-Cl**.
4. Remove 1uL to quantify DNA with nanodrop.

End-repair

1. Kit name: Thermo-Fisher Fast DNA End Repair Kit (Cat. #K0771)
2. Add the following to a clean PCR tube:

DNA	∼3ug ( )
10X End Repair Buffer	5uL
DNA End Repair Enzyme Mix	2.5uL
Water	( )
Total Volume	50uL

3. Incubate at 20°C on lab bench for 5 minutes (do not exceed 20 min).
4. Purify DNA with **90uL** AxyPrep beads. **Elute into 32 uL of Tris-Cl** into a PCR tube.

AxyPrep Mag PCR Clean-up

1. Take AxyPrep Mag PCR Clean-up bead solution out of 4°C. Mix well to homogeneously disperse the beads before use.
2. Add 1.8x volume of beads to sample (18uL per 10uL of sample).
3. Mix beads and sample by pipetting 5 times.
4. Incubate mixed samples for **5 minutes** at room temperature.
5. Place samples onto magnetic plate for ∼ 1 minute, or until solution is clear.
6. Aspirate cleared solution from beads and discard.
7. Hint: Aspirate slowly from the very bottom of the tube and possibly leave a small amount behind to avoid pipetting the beads.
8. Wash beads with 180uL of 70% EtOH. Let sit for 30 seconds before aspirating ethanol and discarding. Repeat for a total of two washes.
9. Allow residual EtOH to evaporate for 1-5 minutes. Do not overdry!
10. Remove samples from magnetic plate. Add appropriate volume (**1-2uL more than you need**) of appropriate elution buffer. Let sit for 1 min to elute DNA.
11. Place samples back on magnetic plate to separate beads from solution.
12. Transfer eluate to fresh tube.

A-tailing

1. Using tube from A-tailing combine:

DNA	32uL
10X ThermoPol Buffer	5uL
10mM dATP	10uL
Taq DNA Polymerase (NEB: #M0267)	3uL
Total Volume	50uL

2. Incubate tube at 72.0 °C for 45 minutes in a thermocycler.
3. Purify with **90uL** AxyPrep beads. **Elute into 13uL of Tris-Cl**. Hint: You can preload the fresh tube with adapter (see: Ligation of adapters).

Adapter Annealing

1. Combine the following in a .2mL PCR tube:

100uM Adapter Oligo 1 (TACCA...)	48uL
100uM Adapter Oligo 2 (ATGATG...)	48uL
50mM $MgCl_2$	4uL
Total Volume	100uL

2. Using thermocycler, heat adapter mix to 95 °C for 10 minutes and then slowly reduce temperature to 20°C over 2 hours (∼ .6 °C/min).

Ligation of adapters

1. To a fresh PCR tube, add the following (add master mix last and mix well):

annealed adapter mix	2uL
(A-tailed DNA)	13uL
Blunt/TA Ligase Master Mix (NEB #M0367S)	15uL
Total Volume	30uL

2. Incubate 60min at room temperature (on bench).
3. Purify with **54uL** AxyPrep beads. **Elute into 21uL of Tris-Cl**.
4. Quantify DNA with nanodrop. You should have .8-2 ug (40-100 ng/uL) of DNA.

PCR1: Himar1 + adapter

1. In a PCR tube combine the following. Hint: Add primers + mastermix to each PCR well first. Then add water and DNA to each well and pipette up-and-down to mix.

Reagent	×1
Q5 Hot Start 2X Master Mix	25uL
10uM adapter primer	2.5uL
10uM transposon primer	2.5uL
Adapter Ligated DNA	800ng ( )
Water	( )
Total volume per tube	50uL

2. Amplify DNA in thermocycler using the following PCR protocol:

1 cycle	30s @ 98 °C
20 cycles	10s @ 98 °C
	30s @ 65 °C
	30s @ 72 °C
1 cycle	2min @ 72 °C
	30 minutes

SPRIselect size selection (230bp-700bp)

1. Thoroughly shake SPRIselect bottle to resuspend beads. Add **27.5uL** of beads to 50uL PCR sample.
2. Mix total reaction volume by pipetting 10 times and incubate at room temperature for 1 minute.
3. Place tube on magnetic stand and allow beads to settle on magnet.
4. Transfer clear eluate to clean tube. Discard tubes with beads.
5. Thoroughly shake SPRIselect bottle to resuspend beads. Add additional **12.5uL** of beads to sample (total volume: 90uL).
6. Mix total reaction volume by pipetting 10 times and incubate at room temperature for 1 minute.
7. Place tube on magnetic stand and allow beads to settle on magnet.
8. Discard supernatant by pipetting from the bottom of the tube.
9. With tube still on magnet, add 180uL of 85% ethanol and incubate at room temperature for 30 seconds.
10. Discard ethanol supernatant by pipetting.
11. Allow residual ethanol to evaporate by leaving tube open on bench for 2 minutes.
12. Remove tube from magnet and add **50uL of Tris-Cl** to elute DNA. Mix by pipetting up and down 10 times and incubate at room temperature for 1 minute.
13. Place tube on magnetic stand and allow beads to settle.
14. Transfer clear eluate to eppendorf tube.
15. Measure samples with Nanodrop. Dilute all samples to between 0.4 - 1.25 ng/uL. Note: Preliminary evidence suggests that Qubit greatly underestimates the amount of DNA at this step (5-10 fold), possibly due to large amounts of ssDNA. Note: Dilution is needed in order to avoid saturating the PCR#2 reaction (which causes PCR bubbles - making BioA useless).

PCR2: Hemi-nested PCR and Addition of Illumina attachment sequences (P5/P7)

1. In a PCR tube combine the following:

Reagent	×1
10uM sol_adapt (matches adapter)	1uL
10uM sol_mar mix (matches Tn)	1uL
Diluted size-selected DNA (0.4 - 1.25 ng/uL)	2uL
Q5 Hot Start 2X Master Mix	10uL
Water	6uL
Total volume per tube	20uL

2. Amplify DNA in thermocycler using the following protocol:

1 cycle	30s @ 98 °C
10 cycles	10s @ 98 °C
	30s @ 67 °C
	30s @ 72 °C
1 cycle	2min @ 72 °C
	15 minutes

3. Purify with **18uL** SPRIselect beads. **Elute into 20uL Tris-Cl**.
4. Quantify DNA with Qubit or qPCR (Nanodrop is NOT reliable). DNA concentration should be between 5 - 20 nM (and must be at least 2nM for sequencing). If concentration is not high enough then increase input to PCR#2. Note: On Agilent Bioanalyzer traces these libraries gave a small peak at about twice the size of the rest of the library (1000bp vs 500bp). This is believed to be due to some non-specific amplicon (likely linear amplification from the adapter sequence). This secondary peak should not affect bulk quantitation via Qubit, Bioanalyzer, or qPCR. Hypothetical ways to eliminate these peaks include increasing cycles of PCR#2. Note: Qubit is faster and easier for quantitation.

Variable Definitions and Setup

The read counts $X_{i,j}$ for each position (i) and each replicate (j) are assumed to be independent for all i, j. For each j, a subset of $X_{i,j}$ are distributed like NE mutants and therefore are identically distributed - but we don't know which subset. Our goal is to find an approximate subset that will have a distribution approximating the null distribution.

Rank-based filter procedure

First we compute the rank of the read counts at each site within each replicate, averaging ties. Call the ranks $r_{i,j}$. Then, for each replicate, compute the mean rank of the *other* replicates (ie leave one out).

$$m_{i,j} = \frac{1}{J-1} \sum_{\tilde{j} \neq j} r_{i,\tilde{j}} \tag{1}$$

Then identify a subset of sites such that the mean ranks are within the expected 40% to 85% range:

$$S_j = \{i : 0.4 < \frac{m_{i,j}}{I} < 0.85\} \tag{2}$$

Finally, assemble the read counts of the null-distributed sites into the set $\bar{X}_j$, which is, by definition, a sample from an approximately null-distributed set of mutants.

$$\bar{X}_j = \{X_{i,j} : i \in S_j\} \tag{3}$$

Thus we have applied a rank-based filter to leave a set of samples that are mutually independent and (approximately) null-distributed.

For simplicity, we index each element of the set $\bar{X}_j$ such that each read count is represented with the variable $Y_{k,j}$ for $k = 1, 2, 3, ..., K$. K is the number of insertion sites after applying the rank-based filter. Therefore $\{Y_{k,j} : k \leq K\} = \bar{X}_j$. $Y_{k,j}$ can now be used for fitting the zero-inflated negative binomial distribution (for ES identification) or for computing the empirical cumulative distribution (for GD/GA identification).

Computation of ES p-value at each insertion site

For simplicity, we drop the j index as we perform identical calulations for each replicate using the corresponding $\bar{X}_j$. The zero-inflated negative-binomial distribution is:

$$N(y; \Theta, r, p) = \Theta \mathbf{1}[y = 0] + (1 - \Theta) \frac{\Gamma(r + y)}{y! \Gamma(r)} p^r (1 - p)^y \tag{4}$$

where $\mathbf{1}[...]$ is the indicator function, $\Gamma()$ is the gamma function. We will use maximum likelihood estimation to fit the parameters. The log-likelihood is:

$$\begin{aligned} l(\Theta, r, p) = z \ln(\Theta + (1 - \Theta)p^r) + (K - z) \ln(1 - \Theta) + \sum_i \ln \left(\frac{\Gamma(y_i + r)}{y_i! \Gamma(r)} \right) \\ + (K - z) r \ln(p) + \ln(1 - p) \sum_i y_i \end{aligned} \tag{5}$$

where K is number of samples and z is the number of samples that are zero (ie $z = \#\{y_k = 0\}$). The gradient is:

$$\frac{\partial l}{\partial \Theta} = z \frac{1 - p^r}{\Theta + (1 - \Theta)p^r} - (K - z) \frac{1}{1 - \Theta} \tag{6}$$

$$\frac{\partial l}{\partial r} = z\frac{\ln(p)p^r(1-\Theta)}{\Theta+(1-\Theta)p^r} + \sum_i \left(\psi(y_i + r) - \psi(r)\right) + (K-z)\ln(p) \tag{7}$$

$$\frac{\partial l}{\partial p} = z\frac{(1-\Theta)p^{r-1}}{\Theta+(1-\Theta)p^r} + (K-z)\frac{r}{p} + \frac{1}{1-p}\sum_i y_i \tag{8}$$

We solve for estimates of the parameters $(\hat{\Theta}, \hat{r}, \hat{p})$ with the L-BFGS-B algorithm (Scipy v1.2.1). To define a "borderline ES" mutant we scale our parameters such that the mean is 5% of the null model but the dispersion $(\frac{1}{r})$ is identical. Define $\tilde{p} = \frac{\hat{p}}{(1-0.05)\hat{p}+0.05}$. Thus, the cumulative distribution for a "borderline ES" mutant is:

$$F(y) = \sum_{\tilde{y}=0}^{y} N(\tilde{y}; \hat{\Theta}, \hat{r}, \tilde{p}) \tag{9}$$

Define a second function:

$$F^L(y) = \sum_{\tilde{y}=0}^{y-1} N(\tilde{y}; \hat{\Theta}, \hat{r}, \tilde{p}) \tag{10}$$

We calculate a continuous p-value by sampling from a uniform distribution between F^L and F:

$$q_{i,j}^{ES} \sim U(F^L(X_i), F(X_i)) \tag{11}$$

where $q_{i,j}^{ES}$ is the p-value for site i, replicate j. We have included the second index (j) to emphasize that we will have a p-value for each replicate.

Computation of GD/GA p-value at each insertion site

We define the empirical distribution function for replicate j as:

$$G_j(y) = \frac{1}{K}\sum_{k=1}^{K} \mathbf{1}[Y_{k,j} \le y] \tag{12}$$

where $\mathbf{1}[...]$ is the indicator function Also define:

$$G_j^L(y) = \frac{1}{K}\sum_{k=1}^{K} \mathbf{1}[Y_{k,j} < y] \tag{13}$$

where $G_j^L(0) = 0$. Note that because $Y_{j,k}$ takes discrete values, $G_j^L(y)$ and $G_j(y)$ will generally differ. To calculate a p-value for site i (replicate j) we sample a uniform distribution bounded by these two values:

$$q_{i,j}^{G} \sim U(G^L(X_i), G(X_i)) \tag{14}$$
